# The shape of things to come: structural insights into how prion proteins encipher heritable information

**DOI:** 10.1038/s41467-022-31460-8

**Published:** 2022-07-13

**Authors:** Glenn C. Telling

**Affiliations:** Prion Research Center, Department of Microbiology, Immunology, and Pathology, Colorado State University, Fort Collins, CO USA

**Keywords:** Prions, Cryoelectron microscopy, Protein aggregation

## Abstract

The prion hypothesis embodies the radical concept that prion proteins contain the necessary information for infectious replication within their shape, thus obviating the requirement for genomic material. Two elegant papers by Hoyt et al. and Manka et al. describing high-resolution structures of infectious prions bring us closer to answering the long-standing question of how different prion conformations produce heritably distinct diseases.

## Determining the structure of infectious prions

Prions cause fatal, incurable neurodegenerative disorders which include human Creutzfeldt Jakob disease, sheep scrapie, bovine spongiform encephalopathy, and the burgeoning epidemic of chronic wasting disease of deer, elk and other cervids. Long thought to have a viral etiology, the causative agents of these diseases display the peculiar ability to replicate and encipher heritable strain information in the absence of genetic guidance. Such extraordinary properties challenge fundamental concepts of inheritance and infection. The prion hypothesis^1^ is contingent on the protean conformational properties of the prion protein (PrP). During prion replication, PrP^Sc^ templates its pathogenic conformation onto host-encoded PrP^C^ in an iterative process that results in the exponential accumulation of infectivity. While the capacity for prions to manifest strain properties initially presented a conundrum, the notion that such information is enciphered by distinct self-replicating PrP^Sc^ conformations is now generally accepted, but based largely on indirect evidence^2,3^.

PrP is attached to the cell surface by a glycosylphosphatidylinositol (GPI) anchor and can be glycosylated at two asparagine residues. While PrP^C^ is monomeric, soluble, and its structure comprises a predominantly α-helical C-terminal domain and a disordered N-terminal region^4^, the aggregating properties and insolubility of PrP^Sc^ presented significant impediments for characterizing its high resolution structure using techniques such as X-ray crystallography and NMR spectroscopy. Prions have long been known to assume the tinctorial properties of amyloid^5^. While previous low resolution studies on recombinant PrP led to a model in which individual PrP molecules spanned the fibril axis to form a parallel in register intermolecular β-sheet (PIRIBS) structure^6^, these in vitro generated amyloids exhibit little or no infectivity. An alternative model proposed that each PrP^Sc^ monomer in infectious prions folds into a four-rung β-solenoid structure^7^. Early studies showed that infectious prion amyloid with high specific infectivity could be purified by detergent extraction and limited proteolysis of diseased brain extracts^8^. Recent developments in cryogenic electron microscopy (cryo-EM) have allowed investigators to characterize the structural properties of these ex vivo purified prions at high resolution. A recent breakthrough study by Kraus and colleagues determined the near atomic structure of a widely characterized hamster-adapted prion strain referred to as 263K^9^. Kraus and co-workers showed that purified 263 K prions contain helical fibrils with each rung composed of hamster PrP monomers (residues ~95–227) stacked in a PIRIBS-based architecture with N-linked glycans and the GPI anchor aligned asymmetrically along one edge of each single helical fibril. Kraus and colleagues envisaged that the twist of these fibers and their attachment to the cell surface by GPI anchorage produced a distorted tethering effect on the membrane surface^9^.

**Fig. 1:**
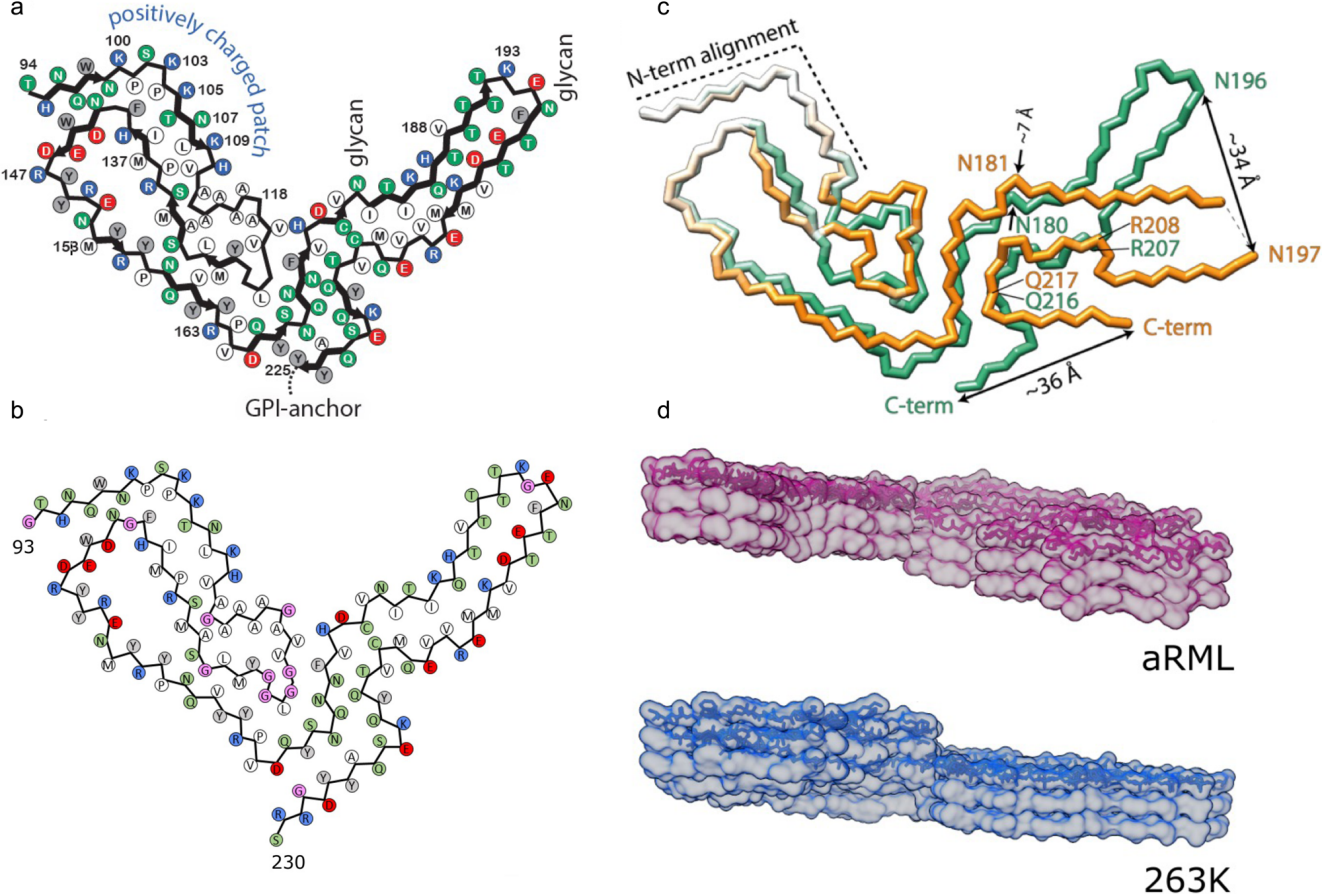
Structures of RML, aRML, and 263 K prions from cryo-EM. Core sequences of PrP subunits in **a** RML filaments and **b** aRML filaments. **c** Comparison of PrP conformations in RML and the 263 K prion fibrils. **d** Lateral views of aRML and 263 K fibrils showing stacks of three rungs. **a** and **c** are adapted from Manka et al.; **b** and **d** are adapted from Hoyt et al.

This discovery raised the question of whether the 263 K hamster prion structure represented an architecture common to all prion fibers, or if different prions have distinct structural properties ranging from PIRIBS-based, to four-rung β-solenoid, or possibly other designs. Moreover, determining the high resolution structural properties of PrP^Sc^ produced by infection with different prion strains offered an opportunity to finally elucidate the details by which heritable information is enciphered by distinct prion conformations. The two current studies in Nature Communications by Hoyt et al. and Manka et al. shed light on these issues^10,11^. Both studies involve cryo-EM analyses of the widely studied mouse-adapted scrapie isolate referred to as RML. However, while Manka and colleagues determined the structure of RML prions isolated from the brains of infected wild type mice, Hoyt and co-workers analyzed the structure of so-called anchorless RML prions (aRML) purified from the brains of infected transgenic mice expressing a mutated form of PrP which lacks the ability to be GPI anchored to the cell surface; anchorless PrP is also glycosylated to a lesser extent than normal mouse PrP^12^. Both studies report near-atomic resolution cryo-EM structures of PrP fibrils. Importantly, RML and aRML have comparable core fibril structures (Fig. 1a, b), and this shared architecture is in turn consistent with the previously reported PIRIBS configuration of hamster 263 K prion fibrils^9^. These findings therefore further challenge the legitimacy of previously postulated models, and increase the likelihood that the PIRIBS architecture shared by hamster 263 K and mouse RML fibrils is a common feature of all mammalian prions.

The complimentary results of Hoyt et al. and Manka et al. provide important cross-validation of the structure of these mouse prions. While resolving the high-resolution structure of aRML was significant in part because the four-rung β-solenoid model was derived from low-resolution cryo-EM analysis of these prions^7^, in isolation they raise doubts about the influences of the GPI anchor and N-linked glycans on the conformational properties of RML. By the same token, Manka et al. used phosphotungstate (PTA) in their prion purification procedure, and these polyanions were found to decorate cryo-EM images of RML fibers leading to similar concerns about the potential for disruption of the RML structure by PTA. The overlapping core architectures of RML and aRML therefore fortuitously demonstrate that the conformation of the amyloid core is unaffected either by the presence or absence of post-translational PrP modifications or by the inadvertent binding of PTA polyanions.

Despite the general similarities between the cryo-EM structures of hamster 263 K and mouse RML prions, pronounced differences become apparent when the conformations of hamster 263 K and mouse RML prion fibrils are aligned. N- and C-terminal lobes are prominent features of both fibers when viewed in cross-section. While the N-terminal lobes of the two strains look relatively similar, the C-terminal lobes, which contain the post-translational modifications, are structurally divergent (Fig. 1c). In profile, the interface between the two lobes is staggered (Fig. 1d). In the case of RML, the N-terminal lobe of one monomer is staggered to align with the C-terminal lobe of the next stacked monomer; in 263 K, the stagger spans almost two monomers in the ladder (Fig. 1d). Manka and co-workers also report that single protofilaments coexist with twisted pairs in their purified RML prion samples. While this is in keeping with previous reports from this lab which argued for the importance of the paired filaments, it seems to be different from purified preparations of 263 K hamster prions that were reported to consist only of single protofilaments. The presence or absence of paired filaments with unpaired counterparts leads to an intriguing framework to explain how diverse PrP^Sc^ glycoform profiles are associated with different strains. In this scheme, strains comprising paired protofilaments are sterically constrained in their ability to accommodate glycans at both asparagines in the C-terminal lobe and are therefore relatively under glycosylated, while strains comprising single protofilaments lack this restriction and incline towards full glycosylation.

Species-specific variations in the sequences of mouse and hamster PrP result in eight amino acid differences within the ordered cores of RML and 263 K. This means that the distinct conformational properties of RML and 263 K fibrils may be attributable to species-specific primary structural differences, distinctive templating properties of these prion strain conformers, or a combination of both. Ultimately, determining the contribution of strain-specific conformational differences requires high-resolution cryo-EM structures of fibrils comprising a fixed amino acid sequence generated by infection with distinct strains, that is infection of one species with multiple prion strains adapted for propagation in that host. New information is already forthcoming. At the time of writing, Manka and colleagues have also reported the cryo-EM structure of a second widely used mouse prion strain called ME7^13^. While fibrils of mouse PrP generated by infection with RML and ME7 share the same underlying PIRIBS architecture, they have markedly different topologies including distinct protofibril crossover distances, differences in the depth and width of the clefts that separate the N- and C-terminal lobes, and variations in the staggered stacking of monomers along their fibril lengths.

As strain and species-specific prion structures continue to be described, it will become increasingly possible to understand how multiple PrP regions coordinate to effect strain-dependent conformational changes of PrP^C^ to PrP^Sc^. While solving the structures of additional well-characterized, biologically cloned, experimentally-adapted rodent prion strains will continue to be significant, high-resolution structural analyses of strains causing naturally-occurring human and animal prion diseases will also be of considerable importance. Elucidating the structural properties of diverse prion strains will ultimately provide a framework for developing novel pharmacological strategies, as well as a means of predicting their potential for interspecies transmission including the risks posed to humans by exposure to emergent prion diseases.
